# Arthroscopic Arthrolysis of the Knee Joint Following Total Knee Arthroplasty

**DOI:** 10.3390/jcm14144917

**Published:** 2025-07-11

**Authors:** Yersin Zhunussov, Yermek Danenov, Galymzhan Alimbek

**Affiliations:** Department of Orthopaedics and Traumatology, International Scientific Center of Traumatology and Orthopaedics, Belbulak Village, Talgar District, Almaty Region 041609, Kazakhstan; dr.danenov@gmail.com (Y.D.); alimbek.galymzhan@mail.ru (G.A.)

**Keywords:** arthrofibrosis, patellofemoral impingement, knee stiffness following total knee arthroplasty, arthroscopic management strategies

## Abstract

**Background:** Arthrofibrosis, mixed contracture, and patellofemoral impingement are frequent complications following total knee arthroplasty (TKA), potentially leading to chronic pain and poor recovery of range of motion (ROM). The comprehensive management of these complications remains challenging and controversial. **Methods**: This study analyzed the outcomes of arthroscopic arthrolysis performed in 27 patients diagnosed with arthrofibrosis, mixed contracture, and patellofemoral impingement post-TKA to evaluate the efficacy of this technique in improving knee function, enhancing ROM, and reducing pain, as assessed by the Knee Society Score (KSS). A total of 27 patients underwent arthroscopic arthrolysis following unsuccessful conservative rehabilitation. The arthroscopic procedure included removal of fibrous adhesions within the suprapatellar pouch, restoration of medial and lateral gutters, and lateral retinacular release of the patella. Intensive physiotherapy and continuous passive motion commenced immediately postoperatively. The mean follow-up period ranged from 24 to 60 months. Pain and functional outcomes were evaluated using KSSs. **Results**: Clinical improvements were evident in 26 cases, with the Knee Society Score rising from a preoperative average of 48 to 86, and pain scores improving from 30 to 41. Only one patient did not experience positive outcomes following the procedure. Arthroscopic arthrolysis appears beneficial for patients suffering from arthrofibrosis, patellofemoral impingement, and mixed contracture post-TKA, significantly improving clinical pain scores and KSS outcomes. **Conclusions**: Further research is recommended to refine specialized surgical instruments and enhance arthroscopic arthrolysis techniques.

## 1. Introduction

According to published data, more than one million patients globally undergo total or unicompartmental knee arthroplasty annually. The primary indication for knee arthroplasty is severe grade III–IV deforming arthrosis, commonly resulting from degenerative joint changes or post-traumatic damage to the articular surfaces of the knee joint [1,2,3,4]. As arthrosis progresses, mixed contractures are observed in approximately 70% of patients, fibrous ankylosis in 15%, and joint stiffness in around 5% [5,6,7]. Both short-term and long-term outcomes of total knee arthroplasty, as reported by the Knee Society and other researchers, indicate excellent results in approximately 65% of cases, good outcomes in 15%, satisfactory results in 8%, and unsatisfactory results in 12% [8,9,10]. Among unsatisfactory outcomes, joint contractures account for about 55% of cases, stiffness accounts for 25%, and component instability or septic complications occur in 20% of cases [11,12,13]. Despite rehabilitation interventions, persistent joint contractures remain in approximately 20% of patients. Consequently, recent studies have increasingly focused on arthroscopic arthrolysis to treat mixed contractures and joint stiffness following total knee arthroplasty [14,15]. The most frequent indications for arthroscopy after knee arthroplasty include arthrofibrosis, patellofemoral impingement, and persistent knee joint contractures. However, a comprehensive literature review revealed a lack of research publications from Central Asian countries addressing this issue, indicating that our study may represent the first investigation conducted in this geographical region.

Therefore, the primary aim of our study is to evaluate the effectiveness of arthroscopic arthrolysis in managing mixed joint contractures and associated pain following total knee arthroplasty in patients without clinical or laboratory signs of infection.

## 2. Materials and Methods

### 2.1. Patient Demographics

Between 2022 and 2024, a total of 27 arthroscopic procedures were performed at the International Scientific Center of Traumatology and Orthopedics in patients who had previously undergone total knee arthroplasty (TKA). The average patient age was 68.4 ± 3.6 years. The mean body mass index was 29.45 ± 4.9. The mean time to onset of symptoms after the initial knee replacement was 33.7 months, ranging from 1 month to 7 years. The duration of symptomatic presentation before arthroscopic intervention averaged 15.6 months (range: 1 month to 5 years). Predominant symptoms included pain in 73% of patients, adhesions or soft tissue impingement in 35%, and stiffness of movement in 20%. The cohort consisted of 26 females and 1 male patient. Mixed contractures were localized predominantly in the right knee joint (n = 19), with the remaining (n = 8) in the left knee. Follow-up duration after arthroscopic surgery following total cemented knee arthroplasty ranged from 2 months to 2 years. At hospital admission, goniometric assessments identified restricted knee joint motion of 15–30 degrees in 14 patients and up to 50 degrees in 13 patients. The primary complaints included limited range of motion and pain at the superior pole of the patella and the projection of the patellar ligament.

### 2.2. Arthroscopic Diagnostics

Patients who underwent arthroscopic procedures had previously completed multiple courses of comprehensive conservative rehabilitation post-total cemented knee arthroplasty, without achieving satisfactory clinical outcomes. Preoperative preparation involved prophylactic antibiotic administration, computed tomography (CT) scans, and laboratory analyses including complete blood counts, biochemical profiles, D-dimer, and C-reactive protein measurements, aimed at excluding periprosthetic infection.

Standard arthroscopic entry points were employed for diagnostic arthroscopy. Typically, a 30-degree arthroscopic camera was introduced through the classic anterolateral portal, facilitating visualization, localization, and assessment of arthrofibrosis types. A medial anterior portal was subsequently established for soft tissue conversion, allowing assessment of implant stability, particularly evaluating the polyethylene liner for stability and wear. During arthroscopic revision, fibrotic strands in the suprapatellar pouch, adhesions between Hoffa’s fat pad and the tibial component, and impingement syndrome involving the inferior medial patellar surface and medial femoral component edge were consistently observed.

To achieve comprehensive visualization, a shaver was inserted through the paramedial portal to remove adhesions and scar tissue. An electrosurgical probe was utilized for precise dissection and release of adhesions between articulating surfaces of the patella, femoral, and tibial components, as well as for vaporization of bleeding tissues. In three cases with dense fibrotic strands in the suprapatellar pouch, a specialized soft tissue rasp was employed under direct arthroscopic visualization. Fibrotic tissues were carefully dissected, progressively increasing knee joint range of motion to achieve maximum flexion of 80 degrees and extension to 180 degrees under direct arthroscopic control. Upon maximal restoration of range of motion, thorough intra-articular lavage was performed using a pressure-controlled aspiration–irrigation pump at 80–100 mmHg with copious 0.9% NaCl solution. Following joint drying, the soft tissue incisions were securely sutured.

Postoperative rehabilitation commenced on the second day post-surgery. Initial rehabilitation included passive mobilization utilizing the “Rebless” system, performing two daily sessions, gradually flexing the knee from full extension (180 degrees) to 90 degrees. Beginning on the third day, the range of passive motion exercises was progressively increased by 5 degrees per session, reaching four sessions daily. Additional rehabilitative measures included lymphatic drainage once daily for up to five days, complemented by physiotherapy sessions that specifically excluded thermal treatments.

### 2.3. Statistical Analysis

All values are presented as the mean ± standard error. Statistical differences were investigated using one-way ANOVA followed by Tukey’s multiple comparison test. The results are presented as mean ± SEM. All statistical analyses were performed using GraphPad Prism Software (v. 9.2; GraphPad, San Diego, CA, USA). Statistical significance was set at *p* < 0.05.

## 3. Results

**Figure 1:**
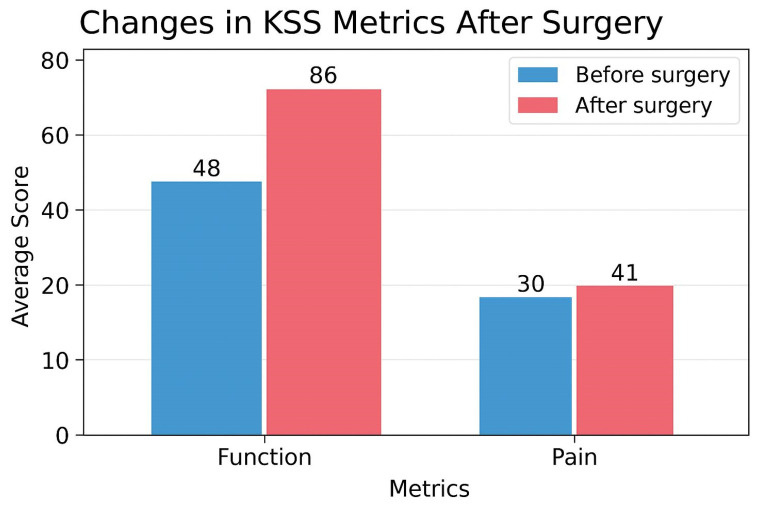
KSSs before and after surgery.

A total of 27 patients’ outcomes were assessed over periods ranging from 2 days to 1 year. The recovery of knee joint range of motion across these follow-up periods is summarized in Table 1. To evaluate the quality of restoration of knee joint motion, the Knee Society Score was utilized. The improvement in KSS before and after surgery is illustrated in Figure 1.

The mean functional amplitude of motion in the knee joint demonstrated substantial improvement from a preoperative average of 153 degrees flexion to 70 degrees at the final evaluation at one year. Pain scores similarly showed improvement, increasing from an average of 30 points preoperatively to 41 points postoperatively. Arthroscopy successfully diagnosed the etiology of symptoms in all cases but one, yielding a diagnostic accuracy of 97.5%. Operative diagnoses included soft tissue impingement beneath the patella consistent with impingement syndrome (43%), hypertrophic synovitis impingement in other regions of the knee joint (15%), anterior knee pain syndrome (10%), polyethylene liner wear (10%), and arthrofibrosis (20%). One-year follow-up results demonstrated that arthroscopic arthrolysis was effective in 85.8% of cases involving arthrofibrosis following total knee arthroplasty. Pain and clinical outcomes did not improve in one patient, accounting for 14.2% of total cases. Taken together, our study shows that arthroscopic arthrolysis is effective in treating patellofemoral impingement following total knee arthroplasty.

*Clinical case*: A 62-year-old female patient underwent total right knee arthroplasty one year prior to her presentation. Initially, postoperative recovery was uneventful; however, eight months after surgery she began experiencing pain along the lateral aspect of the knee joint and a progressive reduction in range of motion, particularly with knee flexion.

**Figure 2:**
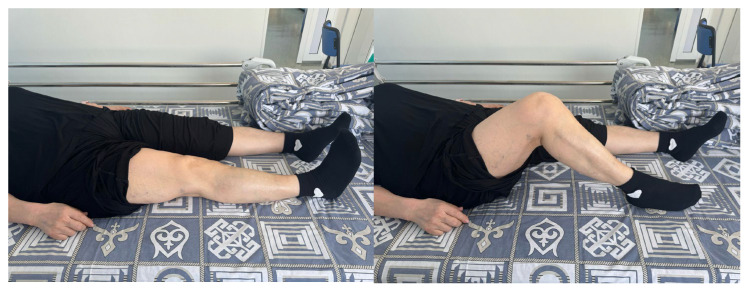
Patient B, 62-year-old female, presenting photograph of the right knee joint upon admission.

Upon clinical examination (Figure 2), pain was localized beneath the patella along its lateral border. Knee flexion was restricted to no more than 110 degrees, while knee extension was complete at 180 degrees.

**Figure 3:**
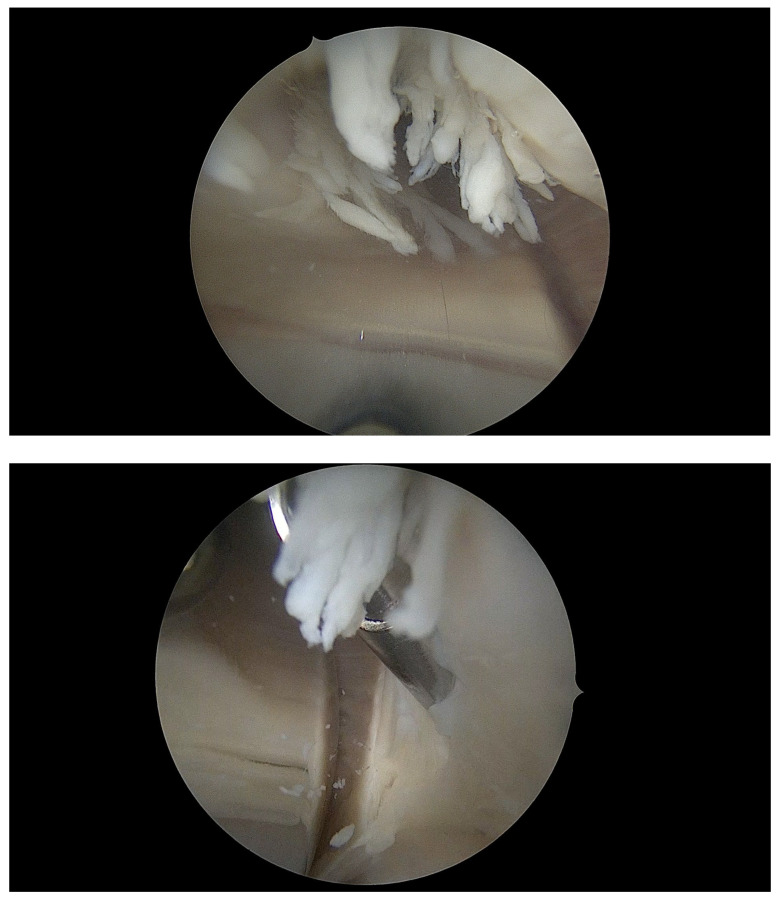

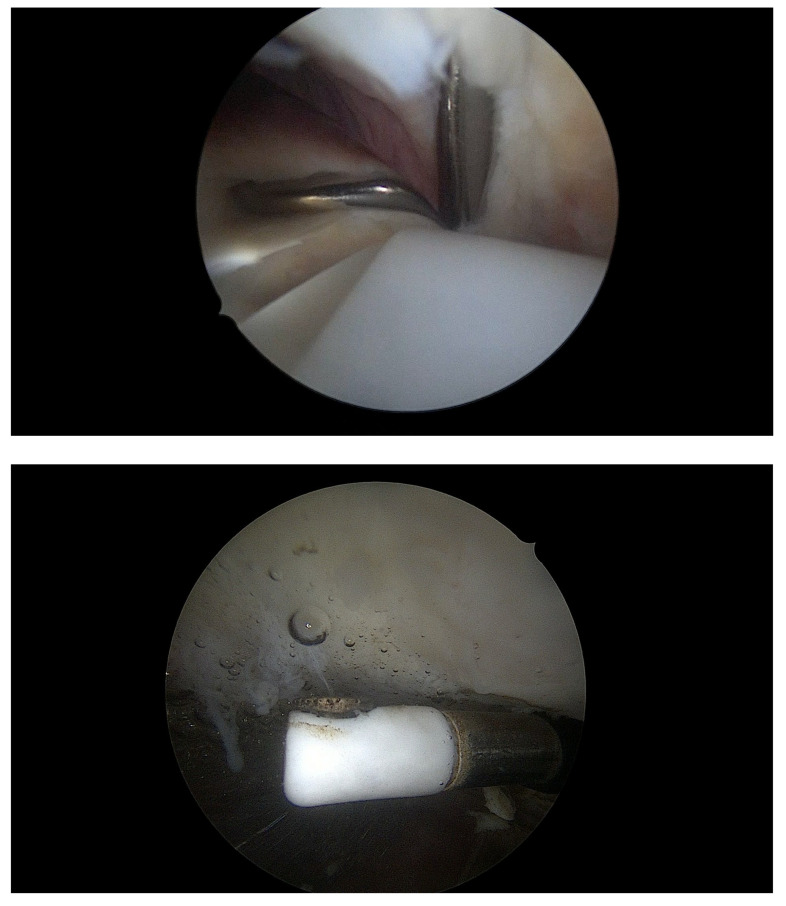
Intraoperative photographic documentation illustrating arthroscopic shaving and vaporization of patellofemoral impingement of the right knee joint in patient B, 62 years old.

Following preoperative preparation and under spinal anesthesia, the patient underwent arthroscopic revision of the right knee joint using standard access with a 30-degree arthroscope. Intraoperative findings (Figure 3) included impingement syndrome between the lateral border of the patella and the lateral aspect of the femoral component, accompanied by marked synovial hyperplasia. The hypertrophic synovial tissue had adhered to Hoffa’s fat pad and was entrapped between the polyethylene insert and prosthetic components during knee flexion.

During arthroscopy, we completely excised the adhesive tissues and treated the impinging patellar surface using a mechanical shaver, subsequently smoothing it via electrocoagulation (“vapr”). Upon reviewing the arthroscopic video recording, we concluded that the cause of the patient’s patellar impingement and pain syndrome was an inadequate rotational alignment of the femoral component, which ideally should have been rotated at least 3 degrees relative to the mechanical axis of the knee joint.

**Figure 4:**
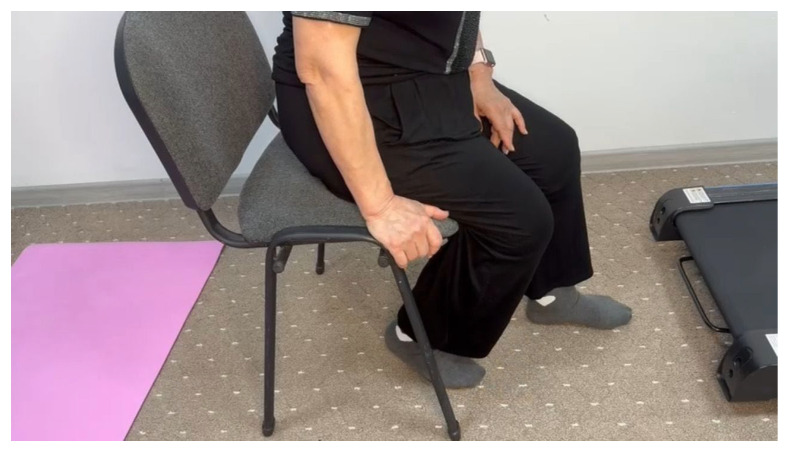
Patient B, 62 years old, demonstrating right knee joint flexion 1-year postoperatively following arthroscopic arthrolysis.

The patient underwent follow-up examinations at 3, 6, and 12 months postoperatively, reporting no further complaints. The range of motion in right knee joint had fully recovered, achieving flexion of 70 degrees and extension of 180 degrees at the 1-year follow-up (Figure 4).

## 4. Discussion

Arthroscopic arthrolysis following total knee arthroplasty (TKA) yielded improvement in 26 of 27 patients in our series, aligning with the high efficacy reported for arthroscopic management of post-TKA stiffness [16]. Notably, one patient did not achieve improvement. This outlier case underscores that persistent stiffness can arise from multifactorial causes that sometimes evade resolution with arthroscopy alone. Biomechanical factors may play a role; for instance, unrecognized component malposition or patellofemoral maltracking can impose a mechanical block to motion that lysis of adhesions alone cannot overcome [17,18]. Prior studies have shown that all cases of stiff TKA in one series had component malrotation on CT, with motion improving only after revision correction [17]. Surgical factors could include incomplete release of dense posterior or extra-articular adhesions during arthroscopy, or unaddressed impingement (such as a fibrous “Cyclops” lesion in the suprapatellar pouch or between components) that continues to limit flexion. In our study, arthrofibrosis and patellofemoral soft tissue impingement were common etiologies; while these typically respond well to arthroscopic debridement (with ~90% success in arthrofibrosis and ~85% in impingement reported) [16], a small fraction may have refractory scar formation or anatomical impediments. Rehabilitation-related factors are also critical. Inadequate postoperative physiotherapy, poor patient compliance with exercises, or insufficient pain control can allow scar tissue to reform and limit the gains of surgery [17]. The literature indicates that a “stiff knee” often results from a combination of surgical and postoperative factors, including suboptimal rehabilitation in an unmotivated patient [17]. Patient-related factors such as a predisposition to exaggerated fibrosis (sometimes termed “arthrofibrosis” tendency), underlying comorbidities (e.g., diabetes, inflammatory conditions), or prior multiple knee surgeries have been associated with higher risk of post-TKA stiffness [17]. It is possible the non-responder had one or several of these risk factors, making the fibrosis less amenable to minimally invasive release. This multifactorial understanding is crucial, as it suggests that the solitary failure in our series might be explained by a convergence of biomechanical issues (perhaps component alignment) and biological scar formation propensity, compounded by any lapses in postoperative rehabilitation.

Arthroscopic arthrolysis is one of several interventions for post-TKA stiffness, and it is important to contextualize its outcomes relative to alternatives like manipulation under anesthesia (MUA), open arthrolysis, and revision TKA. MUA is widely considered the first-line treatment for early postoperative stiffness (typically within 6–12 weeks post-TKA) [19]. Some studies report even larger mean flexion improvements (~30–40°) with timely MUA [17]. The appeal of MUA lies in its simplicity and low invasiveness; however, its success tends to diminish if performed late (beyond 3–4 months post-op) or in the presence of rigid fibrous adhesions. Arthroscopic lysis of adhesions is often the next step for cases unresponsive to MUA or for stiffness presenting later. Our findings reinforce that arthroscopy can effectively address intra-articular scar bands and impinging soft tissue, with a high rate of functional improvement comparable to MUA in appropriately selected patients [17]. Unlike MUA, arthroscopy under direct visualization allows targeted resection of adhesions and can address specific issues like patellar clunk or cement fragments. Reported average gains in knee flexion after arthroscopic arthrolysis range around 25–35° in many series [17,20], and nearly 90% of patients experience meaningful symptom relief [16].

The outcomes of arthroscopic arthrolysis in our series compare favorably with recent reports in the literature. We achieved a significant rate of improved motion or symptoms, which is at the high end of success rates for arthrofibrosis management. Encinas-Ullán et al. reported approximately 90% success for arthroscopic lysis of adhesions in TKA stiffness cases [16], and other series have noted that about 10–30% of patients may experience recurrent stiffness or require re-intervention after arthroscopic treatment [20]. In our follow-up, only ~4% (1 out of 27) had persistent issues, though longer-term observation is needed to confirm the durability of these gains. Notably, timing appears to be a crucial determinant of outcome. Arthroscopic arthrolysis performed within 6 months of the index TKA has been shown to result in superior range-of-motion improvements and functional scores compared to arthrolysis delayed beyond 6 months [17,21]. In our study, most patients underwent arthroscopic release relatively early once non-operative measures failed, which likely optimized results. This aligns with a recent trend favoring early intervention for stiffness: rather than waiting many months in hopes of spontaneous improvement (which is rare in true arthrofibrosis), surgeons now advocate for MUA by 6–12 weeks if ROM is poor, followed by arthroscopic lysis by around 3–6 months if needed [17,19]. Early lysis addresses adhesions while they are less mature and more easily removed, thereby improving the chance of restoring functional motion.

Finally, our discussion would be incomplete without noting the balance of risks. Arthroscopic arthrolysis is minimally invasive, but it is not without complications. The overall complication rate in modern series is low (on the order of a few percent), yet infection in a prosthetic knee is a serious concern. Reports vary on whether knee arthroscopy after TKA significantly raises infection risk; one study observed a prosthetic joint infection rate of ~4% after post-TKA arthroscopy in a mixed group of patients [20], whereas other analyses have not found a statistically higher infection incidence compared to standard TKA patients [22]. In our cohort, we did not encounter any infections or major complications, underscoring that arthroscopic treatment can be performed safely with proper technique and perioperative prophylaxis. This safety profile, combined with the substantial functional benefits, supports the use of arthroscopic arthrolysis as an intermediate step before considering major revision surgery for a stiff TKA. Our results contribute to the growing body of evidence that a structured approach—risk factor optimization, early physiotherapy, timely MUA, and arthroscopic lysis when indicated—can successfully manage the vast majority of post-TKA stiffness cases [17]. Only in the uncommon scenario of persistent stiffness refractory to these measures should invasive options like open release or revision TKA be pursued, as these carry higher morbidity but can still improve outcomes in appropriately selected patients [23,24].

Despite the promising outcomes demonstrated in this study, several limitations must be acknowledged. First, the relatively small sample size limits the generalizability of the findings and precludes robust subgroup analyses based on etiology or comorbidities. Second, the absence of a control group (e.g., patients undergoing alternative treatments such as MUA, open arthrolysis, or revision TKA) limits direct comparisons of efficacy and safety between modalities. While our results are consistent with previous literature, the lack of randomization introduces potential selection bias. Third, the follow-up period, although sufficient to evaluate short- to mid-term outcomes, may not capture long-term recurrence of stiffness or late complications such as prosthetic joint infection or progression to revision surgery.

## 5. Conclusions

Despite to relatively small cohort, our results demonstrated that arthroscopic arthrolysis appears to be a safe and effective technique for managing specific complications of total knee replacement, particularly those involving mixed contracture and patellofemoral impingement syndrome. 

## Figures and Tables

**Table 1:** Range of motion recovery in the knee joint following arthroscopic arthrolysis. The one-way ANOVA followed by Tukey’s multiple comparison test was employed to compare pre-surgery to post-surgery follow-up periods. The results are presented as mean ± SEM. *** *p* < 0.001, **** *p* < 0.0001, and *n.s p* > 0.05.

№	Follow-Up Period	Knee Joint Range of Motion	
		Flexion	Extension
1	Pre-surgery	153 ± 6	175 ± 5 *^n.s^*
2	2 days post-surgery	90 ± 5 ***	175 ± 5 *^n.s^*
3	7 days post-surgery (at discharge)	95 ± 5 ***	180 ± 3 *^n.s^*
4	3 months post-surgery	80 ± 3 ***	180 ± 3 *^n.s^*
5	6 months post-surgery	70 ± 5 ****	180 ± 3 *^n.s^*
6	1 year post-surgery	70 ± 5 ****	180 ± 2 *^n.s^*

## Data Availability

All the data was included in the present article.
